# Will Nigella sativa oil protect parotid glands of rats against cranium gamma irradiation? Histological and immunohistochemical evaluation

**DOI:** 10.1186/s12906-024-04410-8

**Published:** 2024-03-06

**Authors:** Salwa Farid Ahmed, Mostafa A. Bakr

**Affiliations:** Health Radiation Research Dept., National Center for Radiation Research and Technology, Egyptian Atomic Energy Authority, Cairo, Egypt

**Keywords:** Radiation, Salivary glands, Antioxidant, Fibrosis, Nigella sativa

## Abstract

Radiation plays an essential role in treating malignancies. Radiation exposure of salivary glands often results in permanent loss of their functions; therefore, their protection against radiation is crucial. Nigella sativa oil (NSO) is a useful antioxidant against free radicals. The purpose of this study was to investigate the radio-protective effect of NSO on oxidative injury of parotid glands of gamma-irradiated rats. Methods: Twenty-eight male albino rats were divided into four groups (*n* = 7): Group 1: Neither NSO nor radiation, Group 2: Rats received NSO 400 mg/kg, Group 3: Rats received 15 Gy cranium gamma irradiation & Group 4: Rats received gamma irradiation and NSO. Rats were sacrificed two weeks after the last NSO dose. Histological sections of parotid glands were stained with H&E, Masson’s trichrome and anti-TGF-β antibodies. Area percentage of Masson’s trichrome and TGF-β expression was morphometrically examined. Results: Parotid glands of control and NSO groups revealed normal morphology. Gamma-irradiated glands showed loss of normal acinar architecture and slight acinar shrinkage. NSO treatment of gamma-irradiated glands preserved acinar outline and architecture. Masson’s trichrome stained samples revealed trace amounts of collagen fibers in control and NSO groups, and excessive amounts of collagen fibers in gamma-irradiated group, in addition to few collagen fibers for gamma-irradiated glands treated with NSO. Additionally, control and NSO groups showed negative TGF-β expression. Gamma-irradiated group showed high TGF-β expression, while NSO treated gamma-irradiated group showed moderate TGF-β expression. Conclusions: Gamma-irradiation adversely affected parotid glands, and in contrast, NSO seemed to positively counteract this adverse effect.

## Introduction

Neck and head malignancy is one of the most widespread cancers worldwide and causes more than 550,000 cases yearly. Its management includes surgery, chemotherapy, radiotherapy (RT) or combination therapy [1]. Presently, radiation plays an essential role in treating malignancies. Approximately 75% of cancer patients need RT either palliative or curative accompanied with or without chemotherapy [2].

Despite the enormous utility of RT, it also has direct and indirect adverse effects on the environment and the human body. RT harms normal cells and tissues of the body side by side to cancer cells. RT has several side effects for patients such as reduced white blood cells, lowered immunity and production of reactive oxygen species (ROS). Acute radiation syndrome causes loss of appetite, nausea, fatigue, diarrhea, vomiting and decreased white blood cells and immune function. Acute radiation injury can occur at a radiation dose of 1 Gy and is aggravated by increasing the absorbed dose [3, 4]. Radiation exposure of salivary glands often results in an irreversible loss of function that may continue for life with subsequent oral complications and influence quality of life. Xerostomia is a common irreversible postradiotherapeutic complication of neck and head cancers. Therefore, protection of salivary glands against radiation is crucial [5].Parotid gland is more radiosensitive than the submandibular glands. An approximately 50% reduction in parotid gland function has been reported within a few days after low doses of radiation exposure [6].Therefore, our data focused on parotid gland.

Several treatments have been attempted against postradiotherapeutic salivary gland dysfunction such as cytokines [7], cell therapy [8] or antioxidants [9]. Furthermore, natural antioxidants have the ability to scavenge free radicals produced by ionizing radiation in addition to diminishing the complications of oxidative stress [10]. Consequently, it is essential to recognize new radioprotective molecules based on this mechanism.

Nigella sativa (NS) is a dicotyledonous miraculous herb of Rananculaceae with great historical and religious value. It is commonly known as black seed. Seeds of NS contain proteins, alkaloids, fixed oil and essential oil [11]. Nigella sativa oil (NSO) was reported to be a useful antioxidant against free radicals that are responsible for cellular oxidative damage, in addition to having hepato-protective, neuro-protective, antidiabetic, anti-inflammatory and nephro-protective properties [12–14]. NSO acts as a natural free radical scavenger, improving antioxidant status and inhibiting lipid peroxidation-related cellular membrane damage which results in cell death [15].

Moreover, some researches have highlighted the protective role of NS on various organs against exposure to ionizing radiation. In this regard, oral administration of NSO before irradiation restored the normal levels of white blood cells, plasma total protein and globulin concentration that were decreased in response to irradiation. NSO has also shown a great ability to rejuvenate lymphoid follicles of spleen and thymus [16]. Another study examined the radio-protective effect of many agents including NSO against cataract induced by a single dose of cranium gamma irradiation at dose of 5 Gy. Among the used agents, NSO has been shown to be one of the most effective substances in preventing cataract [17]. The same dose of irradiation also induced a significant increment of kidney oxidative parameters including; total oxidant status, oxidative stress index and lipid hydroperoxide with significant reduction of total antioxidant status, paraoxonase and ceruloplasmin compared to control. Orogastric administration of NSO (1 g/kg) for 11 days starting one hour before radiation exposure significantly decreased the oxidative parameters and increased the antioxidative parameters due to its antioxidant effects against radiation induced oxidative damage [18].

Recently, NSO showed a promising protection for salivary glands against radiation exposure. The cephalic areas of mice were exposed to a single dose of 15 Gy irradiation. The treatment group received 0.07 mL/kg of NSO gavage starting three days before irradiation and continued for additional 15 days. Irradiated mice treated with NSO showed weight loss at the start of the treatment with gradual recovery over 30 days in contrast to the irradiated group, which failed to regain weight loss. Irradiated mice treated with NSO exhibited more survival rate compared to irradiated ones. Irradiated mice also showed significant decrease in saliva production on days 3 and 16 compared to control, while NSO treatment significantly increased saliva production to a level similar to control. The submandibular and sublingual glands in irradiated mice treated with NSO revealed a histological morphology closer to the control than the irradiated group. Both submandibular and sublingual glands of NSO treated irradiated mice showed more K5 staining at days 16 and 30 compared to irradiated ones indicating salivary gland stem cells retrieval [19].

Several recent studies have investigated the efficiency of NSO against ROS-mediated disorders; however, no study has investigated the effect of NSO on oxidative stress in the parotid glands of rats exposed to cranial irradiation. Accordingly, the purpose of this study was to investigate the radio-protective effect of nigella sativa oil on oxidative injury in the parotid glands of gamma-irradiated rats.

## Methods

### Ethical consideration and animal grouping

The study was carried out on 8–12 weeks old adult male Wistar albino rats weighing 180 ± 20 g at the time of irradiation. Rats were housed with the same free access to standard rat food and water. The experiment was conducted in compliance with the protocol approved by the Research Ethics Committee of the National Center for Radiation Research and Technology (REC-NCRRT), Egyptian Atomic Energy Authority, under the serial number 26 A / 23. Twenty-eight rats were randomly divided into four groups (*n* = 7); group 1: Control; neither NSO nor radiation were administered, group 2: Treatment control; rats received NSO only, group 3: Radiation; rats were exposed to gamma-irradiation only and group 4: Treatment and radiation; rats were exposed to gamma-irradiation and received NSO.

### Nigella sativa oil administration and gamma-irradiation

Rats of groups 2 and 4 received 400 mg/kg body weight NSO by oral gavage for 15 consecutive days started 24 h before radiation exposure [20].

Before localized irradiation, rats of groups 3 and 4 were anesthetized with an intra-peritoneally injected anesthetic combination of 10% ketamine and 2% xylazine (2:1) (0.12 mL/100 g body weight) [21]. Afterwards, rats were completely immobilized inside a special shield then subjected to a single dose of 15 Gy localized (cranium) gamma-irradiation [22] at a dose rate of 11.07 Gy/min using the Gamma Cell (^60^Co), India at the National Centre for Radiation Research and Technology, Egyptian Atomic Energy Authority, Cairo, Egypt. Following irradiation, rats were returned to the Animal Care Centre of the National Centre for Radiation Research and Technology.

### Euthanasia of animals and histological evaluation

Rats were sacrificed two weeks after the last NSO treatment by an overdose of anesthesia (ketamine). Parotid glands were dissected and fixed in 10% phosphate-buffered formalin for 48 h and then embedded in paraffin. Five micron thick histological sections were prepared and stained with hematoxylin and eosin (H&E) and Masson’s trichrome to assess histological changes and fibrosis. Other sections were immunohistochemically stained with anti-TGF-β antibodies. Ten zones were morphometrically examined from each sample magnified at 400X to measure the area percentage of Masson’s trichrome and TGF-β expression using Leica Qwin 500 software.

### Statistical analysis

Data were subjected to one-way analysis of variance (ANOVA) and expressed as the mean and standard deviation. Multiple range tests were used when differences among groups were significant. Statistical analysis was performed using Statgraphics 18 software, Statpoint Technologies, Inc., 560 Broadview Ave. Warrenton, Virginia 20,182. The significance level was set to *P* ≥ 0.05 for all tests.

## Results

### Hematoxylin and eosin

**Fig. 1:**
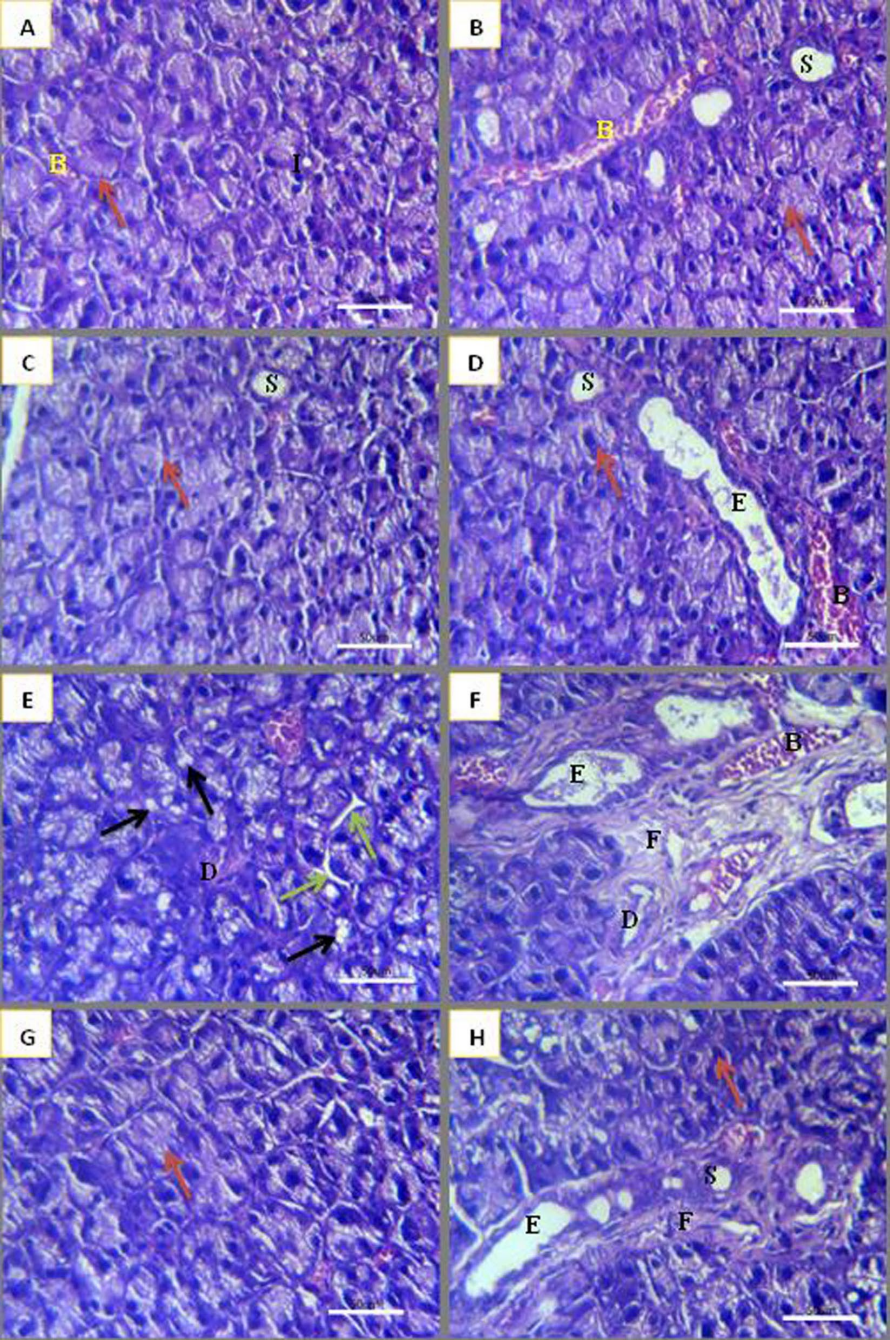
Photomicrographs of parotid glands **(A, B)** control group, **(C, D)** NSO group, **(E, F)** radiation group, **(G, H)** NSO + radiation group. Normal acini (red arrow), vacuoles (black arrow), shrinkage (green arrow), intercalated duct (**I**), striated duct (**S**), excretory duct (**E**), degenerated duct (**D**), blood vessel (**B**). fibrosis (**F**) [**H**. & **E**]

Examination of parotid glands of control and NSO groups revealed normal morphology. The rounded pure serous acini were composed of pyramidal cells with rounded basally situated nuclei enclosing a central lumen. The intercalated ducts were lined by simple cuboidal cells with rounded central nuclei and basophilic cytoplasm. The striated ducts were composed of short columnar cells with central nuclei and eosinophilic cytoplasm. The excretory ducts were lined with pseudo-stratified columnar cells (Fig. 1A-D). Gamma-irradiated parotid glands showed loss of normal acinar architecture and slight acinar shrinkage with massive acinar vacuolization characterized by variable-sized acinar vacuoles. Some striated ducts were partially degenerated while others exhibited complete degeneration. Most striated and excretory ducts were surrounded by excessive amounts of fibrosis (Fig. 1E & F). NSO treatment of gamma-irradiated parotid glands preserved the acinar outline and architecture. Most ducts showed normal morphology, while few of them showed partial degeneration. Some ducts were surrounded by a small amount of fibrosis (Fig. 1G & H).

### Masson’s trichrome

**Fig. 2:**
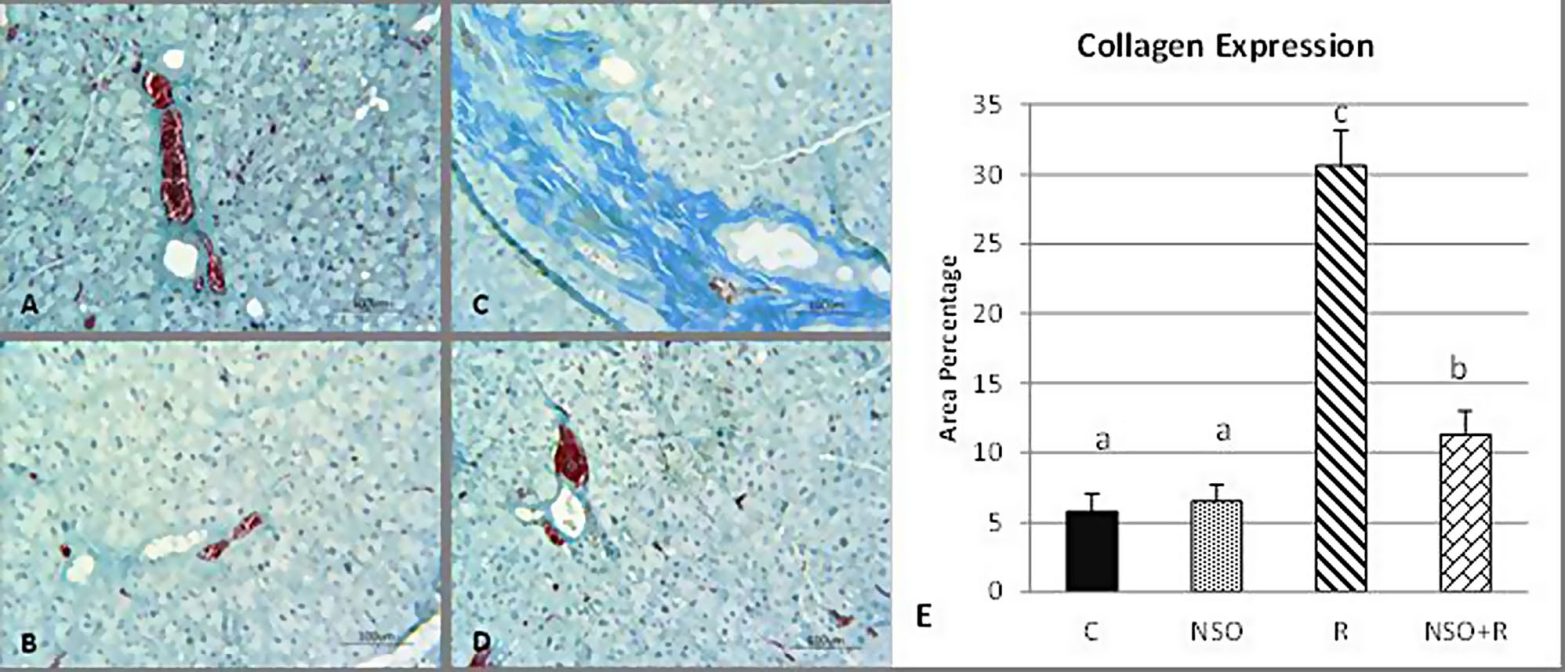
Photomicrographs of Masson’s trichrome-stained parotid glands **(A)** The control group showed traces of collagen around ducts. **(B)** The NSO group exhibited very little collagen around ducts. **(C)** The radiation group demonstrated an excessive amount of collagen around ducts. **(D)** The NSO + radiation group revealed little collagen around ducts. Collagen fibers appeared green‒blue in color. **(E)** Chart representing the area percentage of collagen expression in different experimental groups

Examination of Masson’s trichrome-stained samples revealed trace amounts of collagen fibers in control and NSO-treated groups (Fig. 2A & B), while excessive amounts of collagen fibers were detected in gamma-irradiated parotid glands (Fig. 2C). For gamma-irradiated parotid glands treated with NSO, few collagen fibers were observed (Fig. 2D). Statistically, gamma-irradiated glands exhibited a significantly higher amount of collagen fibers compared to control. NSO treatment of gamma-irradiated parotid glands significantly reduced the amount of collagen fibers compared to gamma-irradiated ones however; it was significantly higher than that of the control (Fig. 2E).

### TGF-β expression

**Fig. 3:**
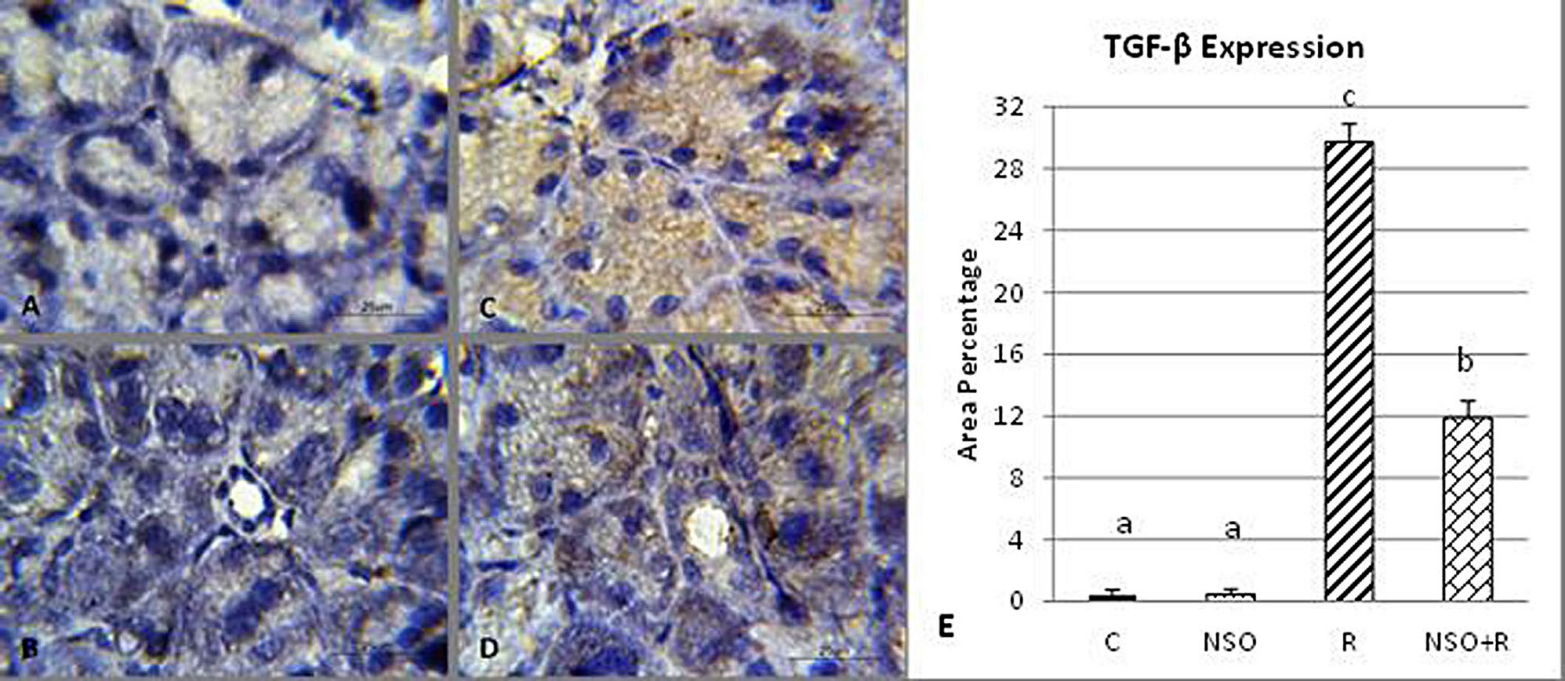
Photomicrographs of TGF-β expression in the parotid glands **(A)** The control group showed almost negative expression, **(B)** the NSO group exhibited almost negative expression, **(C)** the radiation group demonstrated strong expression, and **(D)** the NSO + radiation group revealed moderate expression. **(E)** Chart representing the area percentage of TGF-β expression in different experimental groups

Parotid glands of the control and NSO-treated groups showed negative TGF-β expression (Fig. 3A & B). Gamma-irradiated parotid glands showed massive TGF-β expression (Fig. 3C), while NSO treatment of gamma-irradiated parotid glands showed moderate TGF-β expression (Fig. 3D). Statistical analysis of the area percentage of TFG-β expression is illustrated in Fig. (3 E). The area percentage of TGF-β expression in gamma-irradiated parotid glands was significantly higher than that in all other groups. However, gamma-irradiated parotid glands treated with NSO showed a significantly lower area percentage of TGF-β expression compared to the irradiated group but was still significantly higher than control (Fig. 3E).

## Discussion

Neck and head cancer, including cancers of the buccal cavity in addition to salivary and thyroid glands is the sixth most common malignancy worldwide.The rate of death reaches 350,000 cases out of approximately 600,000 new cases yearly. Radiotherapy is an essential method of cancer therapy either alone or combined with chemotherapy and/or surgery. During RT for malignancies of the neck and head, salivary glands may become unintentionally exposed to radiation, which causes damage, especially if a high radiation dose is used to control cancer [23]. Parotid glands have been suggested to be more [24] or equally [25]vulnerable to radiotherapy compared to submandibular glands, as serous cells show more damage in the form of degranulation, atrophy and pyknosis and at lower doses than mucous and ductal cells [24, 25]. Since parotid gland mainly consists of serous cells, the submandibular gland is a mixed serous/mucous gland, and it might be that the parotid gland is more radiosensitive in terms of saliva production [26].

Our examination of parotid glands 28 days post irradiation revealed loss of acinar outline, severe intracytoplasmic vacuolization of acinar cells, interacinar shrinkage and duct degeneration in addition to fibrosis around ducts. Similar results were reported in the parotid glands 8 h and 30 days following 15 Gy irradiation [27] and submandibular glands 28 days after 18 Gy irradiation [28]. They reported strong vacuolization, many pyknotic nuclei, lysis of acinar and duct cells, degeneration of many granular convoluted tubules and striated ducts epithelial cells, and interstitial edema and fibrotic tissue. Moreover, the number of granular convoluted tubules and acinar cells decreased [27, 28]. In addition, Birer et al. [29] found that radiation treatment (9 Gy) resulted in a significant increase in fibrosis in both salivary glands and tongue. In the same context, irradiated submandibular glands (15 Gy) showed marked acinar cell vacuolization, condensed nuclei, mononuclear infiltration and focal fibrosis 4 weeks post irradiation, in addition to increased collagen deposition [30]. Irradiated human submandibular glands represented similar changes as shown by Nam et al. [31], who reported disorganized acinar and ductal structures, lymphocytic infiltration and high collagen deposition after irradiation.

Morphometric analysis of TGF-β expression revealed a significant increase in irradiated parotid glands in comparison to control and treatment groups. Similarly, Kim et al. [28] found a marked elevation in TGF-β1 protein in submandibular glands, with peak expression on day 28 after 18 Gy irradiation. Additionally, Xu et al. [9] found a strong TGF-β1 expression in the cytoplasm of ductal and some acinar cells of submandibular glands 8 h and 30 days after 15 Gy irradiation with extracellular TGF-β1 expression on the 30th day post irradiation.

Oral administration of NSO prior to gamma irradiation provided a high degree of protection for parotid glands, as evidenced by the absence of acinar vacuolization and duct degeneration in addition to decreased fibrosis. Moreover, it significantly decreased TGF-β expression compared to irradiated glands. These results were parallel to those obtained by Nor-Eldin and Elsayed [32], where administration of NSO improved the cerebral and cerebellar changes induced by exposure to X-ray (8 Gy) with a significant decrease in the number of GFAP-positive astrocytes. Likewise, administration of NSO provided partial protection and improvement of histopathological alterations of parotid gland subjected to fenitrothion intoxication [33]. Mouket et al. [34] demonstrated the radio-protective effect of NSO on salivary glands exposed to total cranial irradiation in terms of a significant reduction in oxidative stress markers and strong antioxidant effects. Additionally, the radio-protective effect of NSO on the irradiated heart was confirmed by Kaplan et al. [35], where NSO significantly decreased TOS and OSI levels in the heart tissue, while TAS levels significantly increased. Moreover, Shahzad et al. [36] found that NSO administration reduced inflammation and collagen deposition in the lungs of ovalbumin exposed rats with a marked reduction in transforming growth factor beta mRNA expression. Furthermore, Majdalawieh et al. [37] found that aqueous extract of NS significantly decreased the levels of the proinflammatory mediators IL-6, TNF-α, and NO by primary macrophages during splenocyte proliferation. Similarly, oral administration of thymoquinone, and NSO component, significantly suppressed the levels of IL-1β, IL-6, TNF-α, IFN-γ and PGE_2_ in an arthritis model in wistar rats [38]. The radio-protective effect of orally administered NSO against whole body gamma irradiation was reported by Assayed [16]on the spleen and thymus and Amin et al. [39]on liver and blood components and they found noticeable regeneration in spleen and thymus lymphoid follicles. NSO administration reduced the hepatic histopathological changes induced by irradiation, where moderate vacuolar degeneration and a clear and non-congested central vein were found. Additionally, it significantly decreased liver MDA, ALT and AST levels in response to irradiation and inhibited the reduction in RBC count and hemoglobin content induced by irradiation [16, 39]. Moreover, Üstün et al. [40] studied the radioprotective effect of NSO on rats’ tongues, which was attributed to the reduction of the oxidative stress index, total oxidant status and lipid hydroperoxides levels, as well as the elevation of paraoxonase levels. They concluded that NSO might be a beneficial radioprotective agent against tissue injury.

## Conclusions

Cranium irradiation (15 Gy) induced histopathological changes in the parotid glands, as evidenced by acinar vacuolization with increased fibrosis around ducts and TGF-β expression. Treatment with Nigella sativa oil preserved the histological structure of the parotid glands, diminished fibrosis around ducts and decreased TGF-β expression. Therefore, it can be used as adjuvant treatment with radiotherapy.

## Data Availability

All data generated or analyzed during this study are included in this published article.

## References

[1] Fazer-Posorske C (2021). A Multidisciplinary Approach to Head and Neck Cancer. J Adv Pract Oncol.

[2] Roach MC, Bradley JD, Robinson CG (2018). Optimizing radiation dose and fractionation for the definitive treatment of locally advanced non-small cell lung cancer. J Thorac Dis.

[3] Taysi S (2008). Melatonin reduces oxidative stress in the rat lens due to radiation-induced oxidative injury. Int J Radiat Biol.

[4] Chang DS, Lasley FD, Das IJ, Mendonca MS, Dynlacht JR (2014). Acute effects of total body irradiation (TBI). Basic Radiotherapy Physics and Biology.

[5] Porter SR, Fedele S, Habbab KM (2010). Xerostomia in head and neck malignancy. Oral Oncol.

[6] Nagler RM (2002). The enigmatic mechanism of irradiation-induced damage to the major salivary glands. Oral Dis.

[7] Lombaert IM, Brunsting JF, Wierenga PK, Kampinga HH, de Haan G, Coppes RP (2008). Cytokine treatment improves parenchymal and vascular damage of salivary glands after irradiation. Clin Cancer Res.

[8] Sumita Y (2011). Bone marrow-derived cells rescue salivary gland function in mice with head and neck irradiation. Int J Biochem Cell Biol.

[9] Xu L (2013). Resveratrol attenuates radiation-induced salivary gland dysfunction in mice. Laryngoscope.

[10] Lobo V, Patil A, Phatak A, Chandra N (2010). Free radicals, antioxidants and functional foods: impact on human health. Pharmacogn Rev.

[11] Tembhurne SV, Feroz S, More BH, Sakarkar DM (2014). A review on therapeutic potential of Nigella sativa (kalonji) seeds. J Med Plants Res.

[12] Hosseini M, Mohammadpour T, Karami R, Rajaei Z, Reza Sadeghnia H, Soukhtanloo M (2015). Effects of the hydro-alcoholic extract of Nigella sativa on scopolamine-induced spatial memory impairment in rats and its possible mechanism. Chin J Integr Med.

[13] Beheshti F (2018). Nigella sativa prevented liver and renal tissue damage in lipopolysaccharide-treated rats. Saudi J Kidney Dis Transpl.

[14] Fanoudi S, Alavi MS, Hosseini M, Sadeghnia HR (2019). Nigella sativa and thymoquinone attenuate oxidative stress and cognitive impairment following cerebral hypoperfusion in rats. Metab Brain Dis.

[15] Ahlatci A (2014). Radiation-modifying abilities of Nigella sativa and thymoquinone on radiation-induced nitrosative stress in the brain tissue. Phytomedicine.

[16] Assayed ME (2010). Radioprotective effects of black seed (Nigella sativa) oil against hemopoietic damage and immunosuppression in gamma-irradiated rats. Immunopharmacol Immunotoxicol.

[17] Demir E (2016). The effects of Nigella sativa oil, thymoquinone, propolis, and caffeic acid phenethyl ester on radiation-induced cataract. Wien Klin Wochenschr.

[18] Alkis H, Demir E, Taysi MR, Sagir S, Taysi S (2021). Effects of Nigella sativa oil and thymoquinone on radiation-induced oxidative stress in kidney tissue of rats. Biomed Pharmacother.

[19] Luff M (2023). Nigella sativa oil mitigates xerostomia and preserves salivary function in radiotherapy-treated mice. Laryngoscope Investig Otolaryngol.

[20] Bayoumi KA, Abdel Fattah A, Gaballah IF (2020). Possible protective potential of atorvastatin and black seed (nigella sativa) oil in amikacin induced nephrotoxicity in adult male albino rats. Egypt J Forensic Appl Toxicol.

[21] De Vasconcelos Catão MH, Nonaka CF, de Albuquerque RL, Bento PM, de Oliveira Costa R (2015). Effects of red laser, infrared, photodynamic therapy, and green LED on the healing process of third-degree burns: clinical and histological study in rats. Lasers Med Sci.

[22] Yang G, Wang Y, Cui Z, Due B, Yin G (2018). Expression and analysis of EPOR after radiation injury of salivary glands in rats. J Odontol.

[23] Singh AK, Pandey P, Tewari M, Pandey HP, Gambhir IS, Shukla HS (2016). Free radicals hasten head and neck cancer risk: a study of total oxidant, total antioxidant, DNA damage, and histological grade. J Postgrad Med.

[24] Liem IH (1996). Evidence for early and persistent impairment of salivary gland excretion after irradiation of head and neck tumours. Eur J Nucl Med.

[25] Valdez IH, Atkinson JC, Ship JA, Fox PC (1993). Major salivary gland function in patients with radiation-induced xerostomia: flow rates and sialochemistry. Int J Radiat Oncol Biol Phys.

[26] Coppes RP, Vissink A, Konings AW (2002). Comparison of radiosensitivity of rat parotid and submandibular glands after different radiation schedules. Radiother Oncol.

[27] Gomes CC, Ramos-Perez FM, Perez DE, Novaes PD, Bóscolo FN, Almeida SM (2013). Radioprotective effect of vitamin E in parotid glands: a morphometric analysis in rats. Braz Dent J.

[28] Kim JH (2016). Protective effects of alpha lipoic acid on radiation-induced salivary gland injury in rats. Oncotarget.

[29] Birer SR (2017). Inhibition of the continuum of radiation-induced normal tissue injury by a redox-active Mn porphyrin. Radiat Res.

[30] Xu L (2016). Simvastatin attenuates radiation-induced salivary gland dysfunction in mice. Drug Des Devel Ther.

[31] Nam K (2016). Post-irradiated human submandibular glands display high collagen deposition, disorganized cell junctions, and an increased number of adipocytes. J Histochem Cytochem.

[32] Nor-Eldin EK, Elsayed HM (2019). The possible radioprotective effects of vitamin E, Nigella sativa oil, and melatonin against X-ray induced early acute changes in cerebral and cerebellar cortices in albino rats: histological and Immunohistochemical. Egypt J Histol.

[33] Korany NS, Ezzat BA (2011). Prophylactic effect of green tea and Nigella sativa extracts against fenitrothion-induced toxicity in rat parotid gland. Arch Oral Biol.

[34] Mouket S, Demir E, Yucel A, Taysi S (2022). Nigella sativa oil reduces oxidative/nitrosative stress in the salivary gland of rats exposed to total cranial irradiation. Drug Chem Toxicol.

[35] Kaplan M (2022). Radioprotective effect of nigella sativa oil on heart tissues of rats exposed to XXXerostomiaXXX. Int J Cardiovasc Sci.

[36] Shahzad M (2010). Suppressive effects of black seed oil on ovalbumin induced acute lung remodelling in E3 rats. Swiss Med Wkly.

[37] Majdalawieh AF, Hmaidan R, Carr RI (2010). Nigella sativa modulates splenocyte proliferation, Th1/Th2 cytokine profile, macrophage function and NK anti-tumor activity. J Ethnopharmacol.

[38] Umar S, Zargan J, Umar K, Ahmad S, Katiyar CK, Khan HA (2012). Modulation of the oxidative stress and inflammatory cytokine response by thymoquinone in the collagen induced arthritis in Wistar rats. Chem Biol Interact.

[39] Amin SM (2023). Radioprotective effect of Nigella sativa Oil (NSO) against radiation-induced hepatic toxicity and haematological alteration in irradiated albino mice. Int J Radiat Res.

[40] Üstün K (2014). Radio-protective effects of Nigella sativa oil on oxidative stress in tongue tissue of rats. Oral Dis.

